# Bulk-independent surface oxide composition controls the electrochemical performance of high-entropy alloys†

**DOI:** 10.1039/d4ta03619k

**Published:** 2024-07-30

**Authors:** Matthias Kogler, Matteo Olgiati, Markus Ostermann, Philipp Rachle, Soniya Gahlawat, Markus Valtiner, Christian M. Pichler

**Affiliations:** a Institute of Applied Physics, Vienna University of Technology 1040 Vienna Austria markus.valtiner@tuwien.ac.at christian.pichler@cest.at; b Center for Electrochemical Surface Technology GmbH 2700 Wr. Neustadt Austria

## Abstract

Multi-element alloys and high-entropy alloys show promising electrocatalytic behavior for water splitting and other catalytic reactions, due to their highly tunable composition. While preparation and synthesis of these materials are thoroughly investigated, the true reactive surface composition is still not well understood, as it may significantly differ from the bulk composition. Precise knowledge and understanding of resulting surface composition is crucial for effective control of the electrocatalytic performance. In this work, low energy ion scattering spectroscopy was applied to determine the surface oxide composition of a series of Ni-based multi-metallic alloys with Mn, Fe, Co, and Cr under alkaline, neutral and acidic conditions. The composition of the surface oxide was investigated with sub-nanometer depth resolution. In electrochemical tests, good catalytic activity was found for the oxygen evolution reaction, although a strong dependence on the selected reaction conditions was observed. The surface composition under OER conditions deviates significantly from the bulk composition. No significant benefit of high entropy alloying compared with binary or ternary alloys concerning catalytic OER performance was found.

## Abstract 2

Low-energy ion scattering revealed the true reactive surface composition of high-entropy alloys, differing significantly from the bulk. No significant enhancement in OER performance was observed with high-entropy alloying compared to simpler alloys.

## Graphical abstract

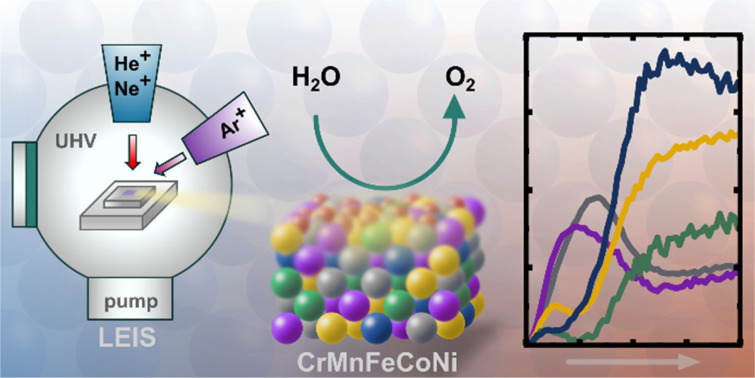

## 1 Introduction

The sustainability driven transformation of the energy, industry, and transport sectors requires long-term energy storage due to the massive expansion of fluctuating electricity generation *via* wind and photovoltaics. As green hydrogen can constitute an essential contribution to long-term energy storage, there has been high interest in developing more active and stable electrocatalysts for the water splitting reaction.^1,2^ While in industrial applications, single-element catalysts such as Pt, Ir, or Ni (or their oxides) are dominating, multi-metal catalysts have generated increasing interest due to their favorable performance concerning activity and stability.^3,4^ By mixing several elements, the catalyst surface composition and properties can be tuned. Based on bi- and tri-metallic compositions, the number of added elements has continuously increased. This approach has gained significant additional attention since high-entropy alloys (HEA) were proposed in 2004.^5,6^ In recent years, HEAs have been increasingly applied as electrocatalysts for water-splitting for both hydrogen evolution reaction (HER) and oxygen evolution reaction (OER).^7,8^ HEAs consist of at least five elements in near-equiatomic concentrations (5–35 at%), and a variety of different combinations have been investigated, based on noble and non-noble metals.^9^ The non-noble metal-based materials are particularly interesting, as the utilization of large amounts of noble metals for water electrolyzers is detrimental from economic and sustainability perspectives.^10,11^ Replacement, of the rare and expensive IrO_2_ for OER or general improvement of the sluggish reaction kinetics and high overpotential are crucial targets in water electrolysis. Especially, Ni/Co-based materials have been demonstrating satisfactory activity for the OER for neutral and alkaline pH conditions.^12–14^ Among the various HEA materials investigated, nanostructured MnFeCoNiCu^15^ showed one of the lowest overpotentials with 263 mV at 10 mA cm^−2^, and even lower charge transfer resistance compared to a commercial RuO_2_ catalyst material. Also, other high entropy compounds, such as multi-element perovskites have been reported.^16^ Although HEAs and other multi-metallic alloys show promising catalytic behavior, the vast majority of studies in this field is focusing on the synthesis, preparation and electrocatalytic benchmarking of those materials, with very basic post-exposure analysis with microscopy or XRD. As recently highlighted, most studies do not consider the stability of the materials, especially the fact that the surface composition of those highly complex multi-element materials and HEAs can potentially differ significantly from their bulk structure.^7^ Segregation or preferential surface accumulation is well known for simpler bi- or tri-metallic systems, however, for electrochemical tests of HEA materials, these factors have been rarely investigated.^17–19^ Even though, precise knowledge and control of the reactive surface composition is of paramount importance and has a major influence on the catalytic activity.

In this study, we combine X-ray photoelectron spectroscopy (XPS) and low energy ion scattering spectroscopy (LEIS), to determine the surface composition of Ni-based materials with increasing complexity, starting from bimetallic materials up to HEAs (Cantor alloy, CrMnFeCoNi). LEIS analysis allowed determining the catalytically active surface layers with close to atomic resolution. The obtained data shows that surface compositions differ significantly from the nominal bulk composition, for all investigated materials. The surface characterization results were related with electrochemical tests for OER under various reaction conditions (acid, neutral, alkaline electrolyte) revealing structure–activity–stability relations. The composition of the surface oxide and its ability to incorporate and stabilize catalytically active sites, mainly determines the catalytic activity for OER. The results include analysis of a great variety of catalytic multi-metallic materials for OER (including steel-like alloys) with outstanding accuracy and detailed insight.

The obtained results demonstrate that the surface composition for multi-metal catalysts deviates significantly from the bulk structure, unravelling the role of individual elements in the formation of the catalytically active surface layers. Furthermore, this generates new insight into the surface dynamics of high-entropy alloys and enables the improvement of future catalyst design. Especially, the supposed beneficial role of the high entropy alloying strategy was shown to be of relatively minor importance. Similar catalytic activity could be even achieved with binary alloys or commodity materials such as Fe-based alloys. The reason for this, is that the surface oxide structure under OER conditions determines the catalytic activity.

## 2 Experimental

All chemicals were used as received without further purification.

### 2.1 Sample preparation

CrMnFeCoNi, CrFeCoNi, CrFeNi, CrCoNi, FeCoNi, CrNi, FeNi and CoNi were prepared *via* vacuum-arc melting with a water-cooled bowl mould as previously described.^20^ All equiatomic samples are reported to be fcc single phased. The samples were used as received. FeCr17.5 wt%, and FeCr17.5Ni10 wt% alloy sheets were purchased from Mateck GmbH, Germany (Purity Fe 99.99%, Cr 99.99%, Ni 99.95%). The sheets were cut into 1 × 1 cm^2^ pieces.

Prior to analysis all samples were ground with P2500 and P4000 sandpaper, polished with 1 μm and 0.1 μm diamond paste to a mirror-like finish, then cleaned in Milli-Q water (18.2 MΩ cm) and ethanol in an ultrasonic bath and used without any further processing.

### 2.2 Electrochemistry

Electrochemical characterization was performed using a PalmSens 4 (PalmSens, Netherlands) potentiostat in a customized cell (redox.me, Sweden) with an active area diameter of 3 mm, a total electrolyte volume of 1.5–2 ml, and a standard three-electrode configuration. As electrolyte 1 M H_2_SO_4_, 1 M Na_2_SO_4_ or 1 M KOH were used. KOH (99.99%), H_2_SO_4_ (98%) and Na_2_SO_4_ (98%) were obtained from Sigma Aldrich. A graphite rod served as counter electrode. Hg/HgO and Hg/Hg_2_SO_4_, respectively, were used as reference electrodes. All potentials were converted to reversible hydrogen electrode using the following equations (*E*_RHE_ = *E*_Hg/HgO_ + 0.059× pH + 0.098; *E*_RHE_ = *E*_Hg/Hg2SO4_ + 0.059× pH + 0.640).

Cyclic voltammetry measurements for 11 cycles with 20 mV s^−1^ between 0.1–0.6 V *vs.* Hg/HgO and 0.5–1.1 V *vs.* Hg/Hg_2_SO_4_ were performed for preconditioning of the surface. Linear sweep voltammetry (LSV) measurements were performed with a sweep rate of 1 mV s^−1^ between 0.1–0.7 V *vs.* Hg/HgO and 0.5–1.17 V *vs.* Hg/Hg_2_SO_4_, whereas the 5th run was used for analysis. For Tafel slope analysis, chronoamperometric (CA) measurements were conducted at given potentials for 150 s, and the current was determined *via* the average of the last 10 datapoints. Before every CA measurement electrochemical impedance spectra were recorded at the given potential, in a frequency range of 1 kHz–100 mHz, with an amplitude of 10 mV. For analysis, PSTrace 5.9 (PalmSens) and the equivalent circuits illustrated in Fig. S5† were used. All potentials were 100% *iR* drop compensated.

Chronopotentiometric measurements of CrMnFeCoNi were conducted in 1 M H_2_SO_4_, 1 M Na_2_SO_4_ and 1 M KOH with a current density of 10 mA cm^−2^. The electrolyte was directly used for ICP-MS analysis.

### 2.3 Low energy ion scattering spectroscopy

Low energy ion scattering spectroscopy was performed using an ION-TOF Qtac^100^ spectrometer (IONTOF, Germany) with ^4^He^+^ 3 keV and ^20^Ne^+^ 5 keV (using a ToF filter) as primary ion at an incident angle of 0° and scattering angle of 145°. The measurement area was 1 × 1 mm^2^ and 0.5 × 0.5 mm^2^. The sputter depth profiling was performed with ^40^Ar^+^ 500 eV, at an incident angle of 59° and a sputter area of 1.5 × 1.5 mm^2^ resp. 1 × 1 mm^2^, and a sputter time of 5 s per cycle. IONTOF Surface Lab 7 was used to process the data. For quantification of Cr, Mn, Fe, Co, and Ni, the measured Ne spectra were background-corrected using a polyline. Reference measurements of monometallic standards were used to determine the exact peak position of the elements. Sample spectra were fitted with Gaussian-typ peaks considering the isotopic distribution of the individual elements, resulting in an asymmetric peak shape for Ni. Intensities were referenced against the respective pure metal signal, measured under equal conditions. The total bulk fraction was set to 1, scaling the individual components accordingly. The H profile was obtained from He 3 keV ToF measurements by extracting the signal for light sputtered ions. As helium is used as primary ion, only hydrogen contributes to this signal.

### 2.4 X-ray photoelectron spectroscopy

X-ray photoelectron spectroscopy (XPS) measurements were performed using a Versa Probe III spectrometer (Physical electronics GmbH) at the ELSA cluster TU Vienna. Monochromated Al K_α_ (1486.6 eV) was used as the radiation source, with the beam diameter set to 200 μm and the beam voltage to 15 kV. The samples were mounted on polymer tape and an E-I neutralization gun was used to balance the charging effect. The samples were sputtered with an Ar ion gun at 1 kV (45°) with a 1 × 1 mm^2^ raster size and a sputter time of 10 s per cycle. Argon 5.0 purity was used for this purpose. Survey scans of all samples were recorded at a pass energy of 140 eV and a step size of 0.5 eV. High-resolution core level spectra were recorded at a pass energy of 55 eV and a step size of 0.05 eV (Cr 2p, Mn 2p, Fe 2p, Co 2p, Ni 2p) and 0.1 (O 1s, C 1s).

CasaXPS was used to process the spectra. Core level spectra were corrected with a Shirley-type background. Metal peaks were fitted with an asymmetric peak shape, while the remaining species were deconvoluted with Gaussian–Lorentzian peak shapes. Auger transitions were obtained from reference measurements and used with all parameters fixed, except for the area.

### 2.5 Inductively coupled plasma – mass spectrometry

Elemental dissolution was evaluated with Inductively Coupled Plasma Mass Spectrometry (ICP-MS, Agilent 7900 ICP-MS, Agilent Technologies). Calibrations were performed prior to any measurements by using a multi-element standard solution (Agilent). The flow of the analyte solution was controlled by a peristaltic pump, which enables a feed rate of approximately 6.6 ± 0.3 mg s^−1^. A Ga-containing (50 ppm) solution was mixed with the analyte and used as an internal standard. For the measurements, samples were diluted to a concentration of 1 mM for all electrolytes.

### 2.6 Scanning electron microscopy

For the determination of the elemental bulk composition Scanning electron microscopy coupled with energy dispersive X-ray spectroscopy (SEM-EDX) was applied Measurements were conducted using a Zeiss Sigma EDVP scanning electron microscope, equipped with an Ametek EDAX analyzer for energy dispersive X-ray spectroscopy (EDX) analysis.

### 2.7 X-ray fluorescence spectroscopy

X-ray fluorescence (XRF) measurements were conducted using an AXIOS advanced spectrometer (Malvern Panalytica, UK).

## 3 Results and discussion

### 3.1 Compositional analysis of the surface region

**Fig. 1:**
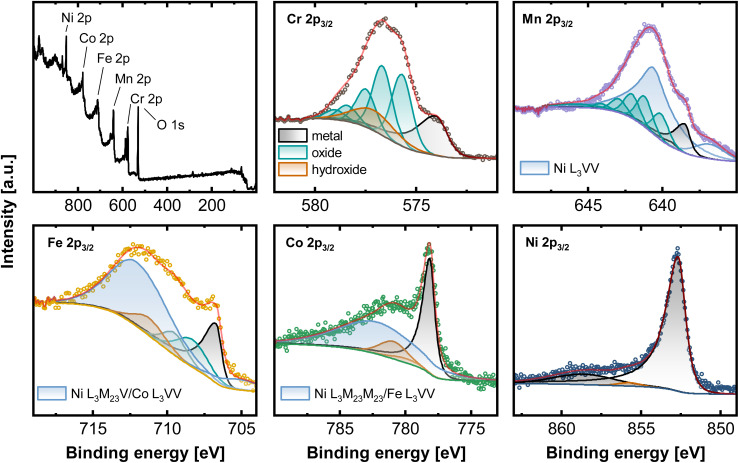
XPS measurements of CrMnFeCoNi recorded on the native oxide after a sputter time of 10 s. Survey spectrum as well as high-resolution spectra of the 2p_3/2_ region of Cr, Mn, Fe, Co and Ni.

Ni based alloys in a series from the Cantor alloy CrMnFeCoNi, CrFeCoNi, CrFeNi, CrCoNi, FeCoNi, CrNi, FeNi to CoNi (always equiatomic element ratio) were investigated systematically. EDX as well as XRF results showed that the bulk compositions for all alloys coincide with the expected equiatomic element ratios (Fig. S1, Tables S1 and S2†). To study the composition of the native alloy surface X-ray photoelectron spectroscopy (XPS) and low energy ion scattering spectroscopy (LEIS) combined with dynamic sputter depth profiling were subsequently applied. Fig. 1 displays the XPS survey spectrum of the native oxide of CrMnFeCoNi, as well as the high-resolution spectra of Cr 2p_3/2_, Mn 2p_3/2_, Fe 2p_3/2_, Co 2p_3/2_, and Ni 2p_3/2_. Spectra were recorded after soft sputtering (Ar^+^ 1 keV, 10 s) for cleaning adventitious carbon.

Deconvolution of the present core level spectra poses a considerable challenge due to convolution of Auger transitions with the 2p region of all major alloying elements, except for Cr, by Ni L_3_VV (Mn 2p_3/2_), L_3_M_23_V (Fe 2p_3/2_), L_3_M_23_M_23_ (Co 2p_3/2_), Co L_3_VV (Fe 2p_3/2_) and Fe L_3_VV (Co 2p_3/2_).^21,22^

One approach to eliminate the Auger contribution is to determine the lineshapes from reference measurements. By recording *e.g.* spectra in the Fe 2p region on a Ni/Co-based alloy, not containing Fe, the pure Ni L_3_M_23_V/Co L_3_VV profile can be established by fitting with reference peak shapes. This can potentially enable the deconvolution of individual components. Nevertheless, this approach is still containing some uncertainty, especially for the exact chemical states due to the pronounced overlap visible in Fig. 1.

Here, the high-resolution Cr 2p_3/2_ spectrum was fitted with three components. Metallic Cr (574.0 eV), hydroxide (577.3 eV), and oxide with a series of 5 peaks (587.8, 576.5, 577.3, 587.4, and 578.8 eV). Mn 2p_3/2_ can be deconvoluted with Mn_met_ (638.5, 639.4 eV), manganese oxide (640.1, 641.2, 642.0, 643.0, 644.1, 645.8 eV) and two synthetic peaks contributing for Ni L_3_VV. The high-resolution spectra of Fe 2p_3/2_ contain three components associated with Fe_met_ (706.7 eV), oxide (708.3, 709.5 eV), and hydroxide (711.2 eV). Two peaks account for the total signal of Ni L_3_M_23_V and Co L_3_VV. Co 2p_3/2_ can be divided into peaks at 778.15 eV (Co_met_), a Co satellite at 787 eV, and hydroxide at 781.0 eV and 780.9 eV. Ni L_3_M_23_M_23_ and Fe L_3_VV are summed in one peak. Apart from a prominent metallic Ni signal at 852.7 eV, Ni 2p_3/2_ presents a satellite peak at 858.9 eV and a small oxide (853.6 eV) and hydroxide component at 855.9 eV.^21,23–25^

This complex evaluation makes it challenging to obtain depth resolved surface compositions of these multi-metal alloys. The information obtained from XPS is an average composition over the first few nanometers of the sample due to the penetration depth of the XPS method.

**Fig. 2:**
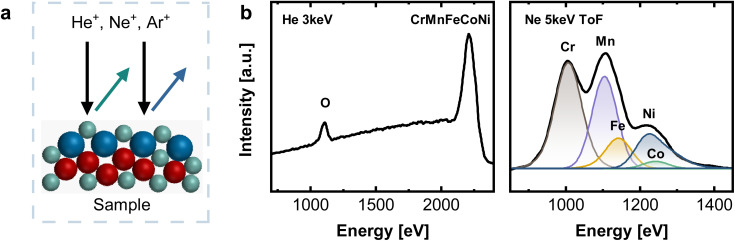
(a) Working principle of LEIS with indicated pathways of primary (black) and scattered (green, blue) ions. (b) LEIS spectra on the native oxide of CrMnFeCoNi recorded with He^+^ 3 keV (left) and Ne^+^ 5 keV (right), after a sputter dose of 2.19 × 10^15^ ions cm^−2^.

Low energy ion scattering (LEIS) is an analysis method that enables true surface sensitivity (single monolayer composition) and avoids the issue of Auger peak overlaps, while sputter depth profiling offers a fully quantitative layer-by-layer decomposition. LEIS (Fig. 2a) relies on noble gas ions being back-scattered at the sample surface. Its unique surface sensitivity results from a combination of short penetration lengths of the noble gas ions due to their low energies between 1–8 keV and the fact that only those ions interacting with the outermost atomic layer possess a sufficiently short interaction time, and resulting single collision scattering. Hence, the outermost surface layer is detected.^26,27^

To gain insight into deeper layers, LEIS can be coupled with Ar^+^ sputtering, and alternating sputter/measurement cycles allow material depth profiles with nearly atomic resolution.

Fig. 2b shows LEIS measurements of CrMnFeCoNi after an Ar^+^ sputter dose of 2.19 × 10^15^ ions cm^−2^, corresponding to an estimated depth of 0.6 ± 0.1 nm. The absence of a C signal at ∼800 eV and a pronounced O peak visible in the spectrum recorded with He^+^ indicate a contamination-free native oxide. Elemental assignment in LEIS relies on differences in scattering energies. A light primary ion such as He does not lead to sufficient peak splitting for clearly distinguishing Cr, Fe, Mn, Co, and Ni (see Fig. 2b) This is indicated by only one pronounced peak at high energies, combining all individual contributions of Cr, Mn, Fe, Co, and Ni. For this reason, measurements were additionally performed with Ne^+^ 5 keV as primary ion, allowing the deconvolution into individual elements (see Fig. 2b and S2†). Time-of-flight (ToF) filtering was applied to reduce the background at low energies resulting from light sputtered ions, as this would overlap significantly with the Cr surface peak. For deconvolution, peak positions of individual elements were fixed to values obtained from reference measurements on the respective monometallic standards.

Combination of XPS and LEIS allows better understanding of the surface structure, especially considering the fact that detailed surface analysis for those materials has not been conducted in great detail. In literature, mostly XPS alone is used, but as just demonstrated, the superposition of Auger transitions, complicates data analysis and the determination of the true nature of the surface structure. By including LEIS as complementary technique, more comprehensive analysis results can be obtained.

**Fig. 3:**
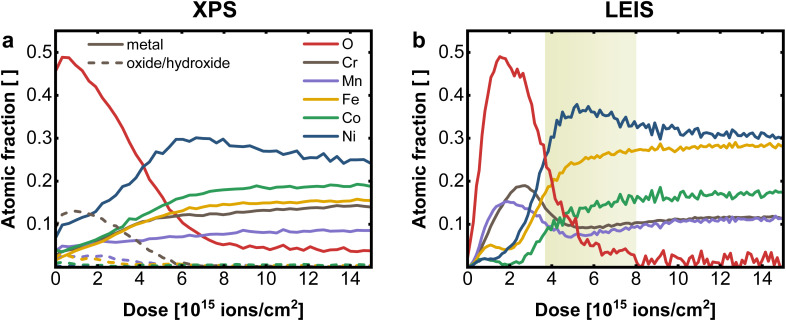
Comparison of sputter depth profiles on CrMnFeCoNi performed with (a) XPS and (b) LEIS. Depletion zone of Mn and Cr in LEIS is highlighted in yellow. A sputter dose of 2.19 × 10^15^ ions cm^−2^ corresponds to an estimated depth of 0.6 ± 0.1 nm (LEIS) resp. 0.7 ± 0.1 nm (XPS).

For comparison of XPS and LEIS analysis, the measured spectra were deconvoluted into their individual elemental contributions as shown in Fig. 1a (XPS) and 2b (LEIS). Combining various spectra at different sputter times or doses allows the establishment of depth profiles, here exemplary for CrMnFeCoNi using XPS and LEIS (Fig. 3). For XPS (Fig. 3a), the atomic fraction from the deconvoluted metal spectra was plotted *vs.* the sputter dose and split up into metallic and oxide/hydroxide contribution, whereas the O contribution was determined by the O 1s peak. LEIS signals are displayed similarly *vs.* the applied sputter dose (Fig. 3b). Here, the atomic fractions are determined by referencing the measured signal against signals from pure metal standards. Oxygen spectra were calibrated using the O profile resulting from measurements with He^+^ to match the total residual of the metal quantification.

From the comparison of the XPS and LEIS profiles for CrMnFeCoNi (Fig. 3a and b), it is evident that even though bulk analysis (EDX, XRF) showed the equiatomic composition of CrMnFeCoNi, the actual bulk values are not reached within the probed thickness. This can mainly be attributed to preferential sputtering, *i.e.*, dynamic change of the surface during depth profiling. This phenomenon can lead to certain elements being enriched during the sputtering process, while others are preferentially removed, depending on the respective sputter yield. This corresponds to the amount of atoms removed upon sputtering at given sputter parameters (sputter species, energy, angle *etc.*). Therefore, in materials with different sputter yields for the individual elements, bulk values cannot be reached. Apart from sputtering parameters, the individual behavior of the elements depends on the elemental mass and on the chemical environment, *e.g.* surface binding energy. Studies have shown, that oxidic materials do not follow the same behavior as non-oxidic specimen, whereas also the complexity of the composite plays an important role, as this also changes the binding properties.^28–31^

The XPS depth profile (Fig. 3a) reveals a significant Ni enrichment with increasing depth accompanied by a decrease in oxygen signal. Furthermore it suggests, that Cr is prevalent on the surface oxide, with only minor contribution of the remaining metals to the oxidic surface region. In XPS, however, by averaging over the first few nm, the concentration of individual elements at the surface tends to be overestimated, resulting in an inaccurate elemental resolution of the surface region. This becomes evident, when compared to results obtained with LEIS (Fig. 3b).

The transition from surface oxide to bulk metal shows a comparable trend with both XPS and LEIS, whereas neither with XPS nor with LEIS the actual bulk values are achieved (*i.e.* preferential sputtering). Again, a significant Ni enrichment in the transition region is observed. Further, especially in the near-surface region, the advantages of LEIS become evident in the first data points.

Both XPS and LEIS suggest Cr is the most abundant element in the oxidic surface layer before leveling off to bulk concentration. Concerning the exact structure of the surface region, LEIS provides, however, more detailed insight.

Apart from Cr, a significantly increased concentration of Mn can be determined in the surface oxide layer, which is not detectable with XPS. However, the maximum of Cr is slightly shifted to deeper layer, indicating that the outer surface oxide is richer in Mn. Both metals show a depletion zone, at the oxide|metal interface after a sputter dose of 4–8 × 10^15^ ions cm^−2^ (depth of roughly 0.8–2.6 nm), coinciding with the Ni maximum, as marked in Fig. 3b. On the terminal surface, however, low amounts of Fe, Co, and Ni are present, markedly below their bulk concentrations. All three elements exhibit a depletion in the oxide-rich region, followed by an increase towards the metallic bulk.

This direct comparison between XPS and LEIS demonstrates the unique surface sensitivity of LEIS, which, coupled with sputtering, enables detailed insight into the elemental distribution over multiple nm, with unprecedented depth resolution. This is especially relevant for the application of those multi-metal alloys in catalysis application. The surface region of CrMnFeCoNi can therefore be described as bilayered structure. The outer layer is the surface oxide/hydroxide, mainly composed of Cr, Mn and only to a smaller extend Fe, Co and Ni oxides/hydroxides. In this outmost Mn/Cr surface oxide small amounts of Ni, Fe and Co can be embedded, which are expected to be relevant for catalytic properties. While Mn is preferentially located at the outmost surface, Cr oxides can be found a few atom layers below, but still in the surface oxide layer. This layer is then followed by a Ni rich partially oxidic interface, and a Cr/Mn depletion zone, before entering the metallic bulk. The surface Ni is formed by an exsolution process from the Ni-rich interface, where Ni can diffuse through the Mn/Cr surface oxide to reach the outer most surface layer. Such mechanisms are well known from passive layer formations in steel and other alloys.^32–34^

**Fig. 4:**
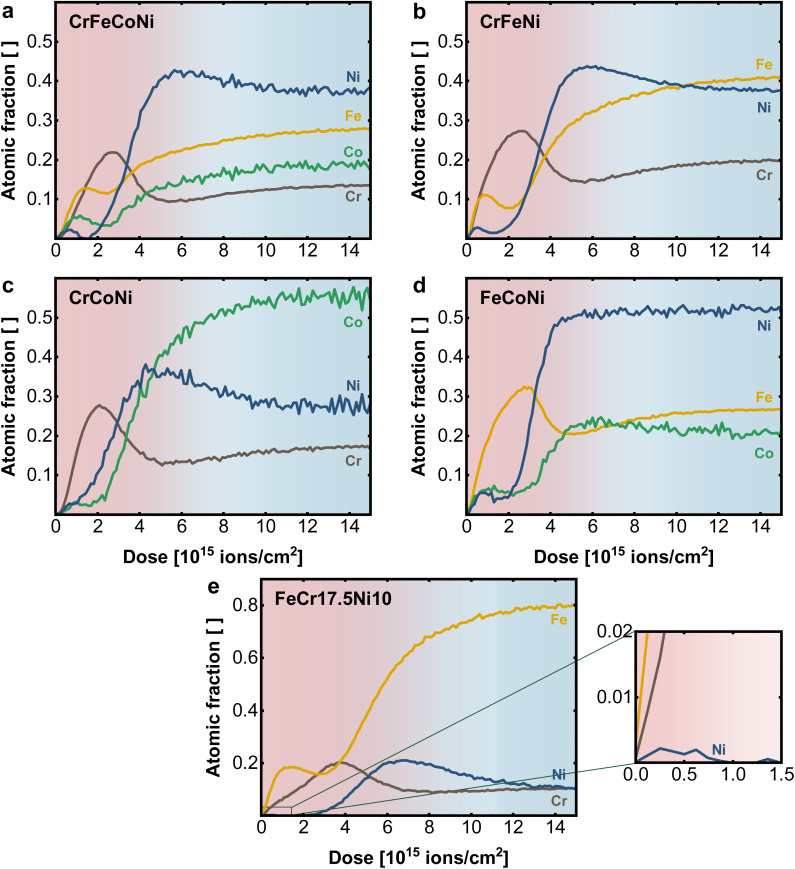
Elemental depth profiles obtained with LEIS on the native surface of (a) quaternary CrFeCoNi, (b) ternary CrFeNi, (c) CrCoNi and (d) FeCoNi and (e) FeCr17.5Ni10, with enlarged surface region.

To investigate the role of the individual alloying components in the material surface, LEIS depth profiles of binary, ternary, and quaternary Ni-based equiatomic alloys were performed (Fig. 4 and S3†). A comparison of the elemental profile of quaternary CrFeCoNi in Fig. 4a with the quinary CrMnFeCoNi (Fig. 3b), which differ only in the absence resp. presence of Mn in the alloy reveals the role of Mn in the oxide layer. While Cr and Ni show comparable behavior, the atomic fraction of Co and especially Fe are significantly increased in the surface oxide layer of the quaternary alloy. This suggests that Mn competes with Fe for presence in the outermost surface layer, as previously shown.^20^

Fe plays nearly the same role in the CrFeCoNi alloy as Mn in CrMnFeCoNi, with a slightly earlier concentration rise compared to Cr, indicating preferential presence in the outer most termination layer. Once more, distinct exsolution of Co and in particular Ni can be observed. It must be remarked again that a sputter ion dose of 2 × 10^15^ ions cm^−2^ indicates a depth of 0.6 ± 0.1 nm in the surface layer. The whole graphs with 15 × 10^15^ ions cm^−2^, therefore, show the elemental distribution over an estimated depth of 3–5 nm.

When analyzing the ternary alloys in more detail, the role of Fe and Cr can be explored further by successively removing one element from the compound. In the absence of Co, CrFeNi exhibits (Fig. 4b) a higher Cr and lower Fe content in the surface oxide layer, compared to the quaternary alloy while maintaining a qualitatively similar profile. Comparing Cr and Fe in separated alloys CrCoNi (Fig. 4c) and FeCoNi (Fig. 4d) shows, that Cr and Fe are in each case the main components of the oxide. However, while the qualitative behavior of Cr does not differ significantly in between alloys, the Fe maximum is moved deeper into the material (at higher sputter dose) and shows a shifted and more pronounced depletion zone at the metal|oxide interface.

The available data can be explained by the different affinity of the alloying elements for oxygen. A general trend is that the more oxophilic elements are agglomerated in the surface oxide layer. While in the quinary alloy, Mn is found in the outermost regions, a comparable structure is formed in the Cr/Fe-containing alloys, which is dominated by Fe on the outer surface, taking the place of Mn in its absence. The profiles further suggest that in general Cr forms a stabilizing layer between the terminal oxide and the Ni enriched metal interface, while Fe, Co and Ni diffuse towards the top of the oxide surface. Here, the bulk composition plays an important role in determining the surface oxide structure, as more oxophilic elements such as Mn, Cr or Fe tend to accumulate at the surface while for Ni and Co only smaller amounts are visible on the surface. In addition, the bulk can serve as a reservoir by replenishing lost/deactivated electrocatalytically active Ni or Co sites *via* a diffusion process from the bulk towards the surface.^34^ A comparable elemental behavior to the multi principal element alloys can be also found for steel-like reference samples such as FeCr17.5Ni10 in Fig. 4e and FeCr17.5 (Fig. S3d†). The Cr maximum coinciding with the Fe depletion zone in the native oxide layer supports the stabilizing effect of Cr, while even at low Ni-bulk concentrations (10 at%) trace amounts are found on the terminal surface (Fig. 4e insert).

### 3.2 Electrocatalytic behavior

With the help of LEIS sputter depth profiles, it could be determined that the surface structure of the investigated materials deviates strongly from the bulk, whereas especially Ni is present in low concentrations at the terminal surface for all alloys. Given the catalytic effect of Ni for alkaline OER, this has a significant influence on electrocatalysis, as the catalytic process takes place at the outermost atomic layer. Low concentration (and thereby finely distributed) surface Ni, can have remarkable catalytic effects.

**Fig. 5:**
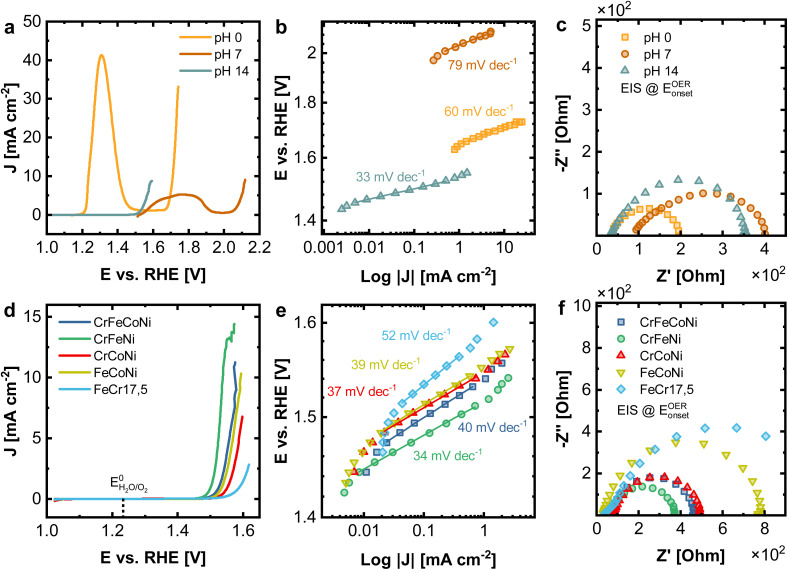
(a–c) Electrochemical characterization of CrMnFeCoNi under different reaction conditions (1 M H_2_SO_4_ (pH 0), 1 M Na_2_SO_4_ neutral (pH 7) and 1 M KOH alkaline (pH 14)) as well as (d–f) CrFeCoNi, CrFeNi, CrCoNi, FeCoNi and FeCr17.5 (all 1 M KOH). (a and d) LSV experiments, (b and e) Tafel plots with obtained Tafel slopes, (c and f) EIS spectra performed at the respective OER onset potential.

To study the electrochemical performance for oxygen evolution reaction (OER), under different catalytic reaction conditions, measurements were performed in acidic (1 M H_2_SO_4_), neutral (1 M Na_2_SO_4_), and alkaline (1 M KOH) electrolyte. Fig. 5a displays the linear sweep voltammetry (LSV) measurements corresponding at different pH values of 0, 7 and 14 for the Cantor alloy. All potentials are given *vs.* the reversible hydrogen electrode (RHE). While for CrMnFeCoNi in acidic and neutral conditions, strong peaks attributed to metal oxidation/dissolution are visible, no signs of instability can be observed in alkaline solution. This is emphasized as well by the cyclic voltammograms (Fig. S4†). Additionally, a strong shift in onset potential for the OER compared to alkaline solutions (1.53 V) is seen in acidic medium (1.65 V), whereas this is even more pronounced in neutral conditions (2.03 V). These high onset pentials are mirrored in the Tafel plots (Fig. 5b), derived from chronoamperometric measurements at given potentials in the OER region. CrMnFeCoNi exhibits a Tafel slope of 60 mV dec^−1^ in acidic conditions, increasing to 79 mV dec^−1^ in neutral medium. However, in alkaline solution, an exceptionally low Tafel slope of 33 mV dec^−1^ is achieved, demonstrating good catalytic activity, suggesting an altered reaction pathway.

In Fig. 5c the impedance spectra for all three conditions recorded at the OER onset potential are displayed. The equivalent circuit used for fitting can be found in Fig. S5a,† where *R*_S_ stands for the electrolyte and *R*_CT_ for the charge transfer resistance. In acidic conditions, for *R*_S_ a value of 34.4 Ω and for *R*_CT_ 166.5 Ω is observed. In neutral medium, this increases to 87.1 Ω (*R*_S_) and 336.2 Ω (*R*_CT_), respectively, whereas in alkaline solution, 34.0 Ω (*R*_S_) and 334.8 Ω (*R*_CT_) obtained. The reason for the comparably small resistance but poor OER performance under acidic conditions can be explained by significant dissolution of the material, while it is stable under alkaline conditions due to a stable passive film formation.

To confirm passive film formation and characterize the material stability in greater detail, chronopotentiometric measurements were carried out at 10 mA cm^−2^ for CrMnFeCoNi in 1 M H_2_SO_4_, 1 M Na_2_SO_4_, and 1 M KOH (Fig. S6†). For comparison, measurements of FeCr17.5 and FeCr17.5Ni10 in 1 M KOH were also performed. Subsequently, the solution was analyzed with ICP-MS to determine metal dissolution. While OER can be observed at this current density in neutral and alkaline conditions, solely metal dissolution occurs in acidic medium, as also clearly visible by a large oxidation peak in the CV (Fig. S4†). In acidic electrolytes, strong dissolution equally distributed over all alloying elements can be observed. In neutral conditions, preferential dissolution of Cr, Co, and Ni is occurring. This can be attributed to incomplete Cr passive film formation under these conditions, whereby it was shown that Mn can have an additional negative effect on the stability (Fig. S7†).^20,35^ Already indicated by the electrochemical measurements, stable behavior is observed in alkaline medium. No significant metal dissolution occurs under these circumstances. As already mentioned, the stability in alkaline solution is attributed to the fact that a passivating oxide/hydroxide layer is formed under those reaction conditions, preventing metal dissolution. This formation, particularly of hydroxides, can be confirmed by additional characterization of selected alloys after chronoamperometric treatment (10 mA cm^−2^) in 1 M KOH. XPS analysis of CrMnFeCoNi, CrFeCONi, CrCoNi and FeCoNi (Fig. S8–S11†) reveal the strong presence of hydroxides among all samples. By performing LEIS He 3 keV Tof measurements, increased H signal can be found after electrochemical treatment in 1 M KOH, when compared to the native oxide, confirming the formation of hydroxides on the surface (Fig. S12†). This displays the underlying transformation of oxides to oxyhydroxides under alkaline OER conditions, as previously reported.^36,37^

Before performing the measurements, the always present surface contaminations require a cleaning step (gentle sputtering for very short time). This cleaning step can potentially alter slightly the structure of surface hydroxides, as they are formed on the outmost surface layer. While this does not influence the fundamental conclusions of the analysis, the amount of hydroxides might be slightly underestimated, when quantifying them.

In order to compare the electrocatalytic activity of the different alloys, 1 M KOH was selected as the standard electrolyte, as the most promising results can be expected in this electrolyte.

In Fig. 5d–f the electrochemical comparison of quaternary CrFeCoNi with the ternary alloys and FeCr17.5 (resembling standard stainless steel) as a reference is made. The results of the binary alloys and FeCr17.5Ni10 (as additional steel-like reference), as well as the results for EIS can be found in Fig. S13 and Table S3.† CrFeNi shows the lowest onset potential (1.51 V, Fig. 5d), and with 33 mV dec^−1^ also the shallowest Tafel slope (Fig. 5e), comparable to CrMnFeCoNi. CrFeCoNi, FeCoNi and CrCoNi show slightly higher onset potentials with respect to the OER (1.53 V, 1.53 V and 1.54 V), as well as slightly steeper Tafel slopes of 40 mV dec^−1^ (CrFeCoNi), 39 mV dec^−1^ (FeCoNi), 37 mV dec^−1^ (CrCoNi). FeCr17.5 shows significantly reduced catalytic activity in these experiments (1.56 V and 52 mV dec^−1^).

The Tafel plot allows insights into the nature of the reaction kinetics and rate-determining steps. A Tafel slope of 30 to 40 mV dec^−1^ indicates a high initial coverage of M–OH species, with M being a free metal site. The rate-determining step can be assumed to involve the adsorption and conversion of free OH^−^ on the initially formed M–OH species. Higher Tafel slopes of approx. 60 mV dec^−1^ indicate a change of rate-determining step, involving an increased coverage of M–O species.^38^ The results hence suggest that the rate-determining steps in the reaction pathway are changing, depending on the used alloy. Additionally, the investigated alloys form a passive oxide/hydroxide layer under alkaline conditions (as shown in Fig. S8–S12†), thus increasing the stability of the surface in general. Under neutral or acidic conditions, this passivating oxide layer is less stable, leading to significant material dissolution.

**Fig. 6:**
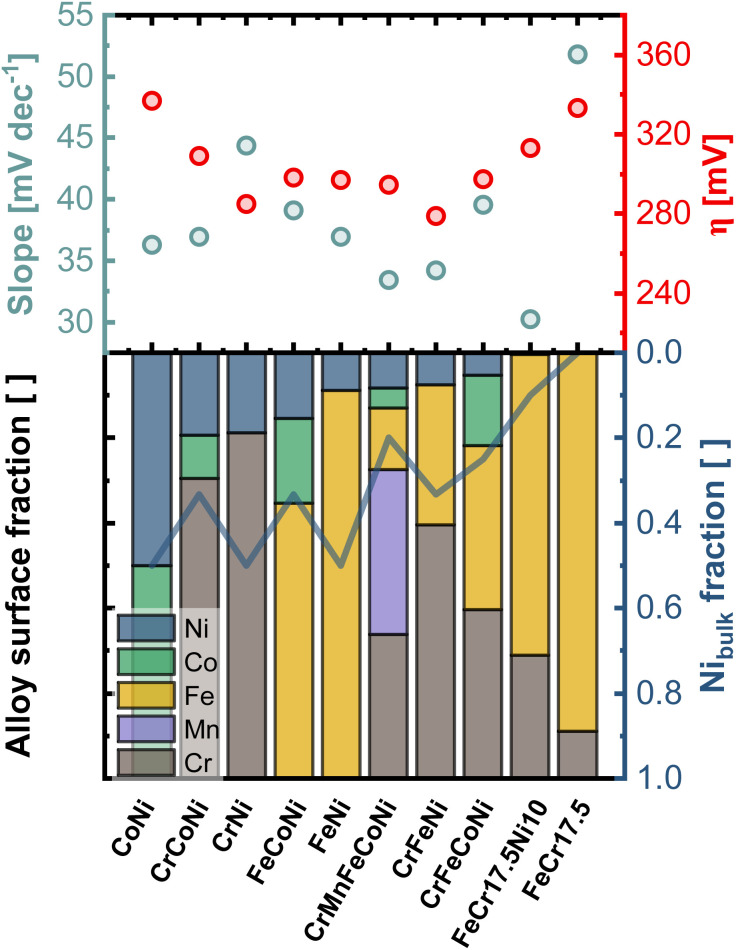
Comparison of the electrocatalytic activity of all investigated alloys, expressed *via* the Tafel slope and OER onset-overpotential (*η*), and the composition in the first nm of the surface region, with indicated Ni bulk fraction (blue line). Sorted by descending amount of Ni in the outermost layers.

Fig. 6 compares the electrocatalytic activity towards OER with the composition of the surface region determined from the first nm of the surface oxide (integration over a dose of 2 × 10^15^ ions cm^−2^). The samples are sorted with respect to the Ni amount in the outermost layers, whereas the line is indicating the Ni-bulk fraction. While a particularly high amount or no Ni in the outermost layer give rise to high onset potentials, similar lower values can be found for the remaining alloys. CrMnFeCoNi shows a low Tafel slope of 33 mV dec^−1^, which is due to the facilitated formation of the oxide/hydroxide layer on the generated bilayer structure.

The importance of Ni as alloying component is illustrated by comparing FeCr17.5 and FeCr17.5Ni10. The binary Fe-based alloy, without Ni shows poor overall performance. In contrast, in FeCr17.5Ni10 Ni proves to be crucial for the formation of the active sites in the oxide layer, as shown in Fig. 4e. Regarding the kinetics, the importance of Ni is highlighted even more. Although, only trace amounts of Ni are found at the outermost surface (as shown in Fig. 4e insert), this structure results in an exceptionally good Tafel slope of 30 mV dec^−1^ (Fig. S13b†), highlighting that the oxide decoration with Ni is central, and not the bulk composition, under OER conditions.

In particular, the significantly improved behavior of FeCr17.5Ni10, can be explained by the trace amounts of Ni in the surface oxide (Fig. 4e insert). As mentioned previously, the highly dispersed Ni species located in the Cr based surface oxide appear to be highly beneficial for OER activity.

## 4 Conclusions

Electrochemical data, including LSV, Tafel slope analysis, and EIS, show that besides the CrMnFeCoNi HEA, also other less complex materials such as CrFeCoNi, or FeCr17.5Ni10 exhibit similarly good, or better, electrocatalytic performance with respect to OER, due to the presence of catalytic elements on the surface oxide. From these results it can be concluded that it is not the design of the bulk material that is decisive, as in the case of HEAs, but the structure and surface bound species of the resulting thin-film oxide. Specifically, combination of the outermost, catalytically active surface species, dispersed on top of the stabilizing passive oxide film, are crucial for improving the overall performance towards alkaline OER. Here, the best performance was achieved with the cheapest material containing nickel, namely the FeCr17.5Ni10 alloy (resembling a standard stainless steel). In this view, design of expensive multielement alloys appears to offer little to no benefit under OER conditions. Instead, embedding of surface reactive catalytic centers on top of a passivating oxide provides both high reactivity and stability in alkaline media.

These findings have significant implications for the future study and development of HEAs and other multi-metallic catalyst materials for OER. The surface oxide design must be tuned and optimized for developing improved catalysts not the bulk.

## Data availability

The raw and processed data required to reproduce these findings are available from the corresponding author upon reasonable request or *via*https://researchdata.tuwien.ac.at/.

## Author contributions

C. M. P and M. V. conceptualized and supervised this work. M. K. performed LEIS measurements. M. K. and P. R. performed electrochemical experiments. M. Olgiati performed ICP-MS measurements. M. Ostermann performed SEM-EDX measurements. S. G. performed XPS measurements. M. K., M. V. and C. M. P. wrote the manuscript. M. V. provided funding for this work. All authors participated in discussions and corrections of the manuscript.

## Conflicts of interest

There are no conflicts to declare.

## Supplementary Material

TA-012-D4TA03619K-s001
