# Supplementary material for: Bulk-independent surface oxide composition controls the electrochemical performance of high-entropy alloys
Source: J Mater Chem A Mater. 2024 Jul 30;12(34):22565–75. doi: 10.1039/d4ta03619k (PMC11348829; doi:10.1039/d4ta03619k)
Supplement: TA-012-D4TA03619K-s001 [file TA-012-D4TA03619K-s001.pdf]

Supplementary Information (SI) for Journal of Materials Chemistry A.
This journal is © The Royal Society of Chemistry 2024

Supporting Information

Bulk-independent surface oxide composition controls the electrochemical performance of high-entropy alloys

Matthias Kogler[a,b], Matteo Olgiati[a,b], Markus Ostermann[b], Philipp Rachle[a], Soniya Gahlawat[a,b], Markus Valtiner[a,b], and Christian M. Pichler*[a,b]*

[a] *Institute of Applied Physics, Vienna University of Technology, 1040 Vienna, Austria*

[b] *Center for Electrochemical Surface Technology GmbH, 2700 Wr.Neustadt, Austria*

Table S1: SEM-EDX analysis of Ni-based alloys. Composition considering only metal components.

Sample	Cr (at%)	Mn (at%)	Fe (at%)	Co (at%)	Ni (at%)
CrMnFeCoNi	20.3 ± 2.7	20.3 ± 2.6	20.5 ± 2.9	19.9 ± 3.1	18.9 ± 3.5
CrFeCoNi	25.5 ± 2.7	--	25.5 ± 2.9	25 ± 2.8	24.1 ± 3.1
CrFeNi	34.2 ± 2.4	--	33.7 ± 2.9	--	32.1 ± 3.1
CrCoNi	33.9 ± 2.5	--	--	33.7 ± 2.7	32.4 ± 2.7
FeCoNi	--	--	33.9 ± 2.5	33.7 ± 2.3	32.4 ± 2.8
CrNi	52.4 ± 2	--	--	--	47.6 ± 2.6
FeNi	--	--	52 ± 2.3	--	48 ± 2.7
CoNi	--	--	--	50.3 ± 2.1	49.7 ± 2.2

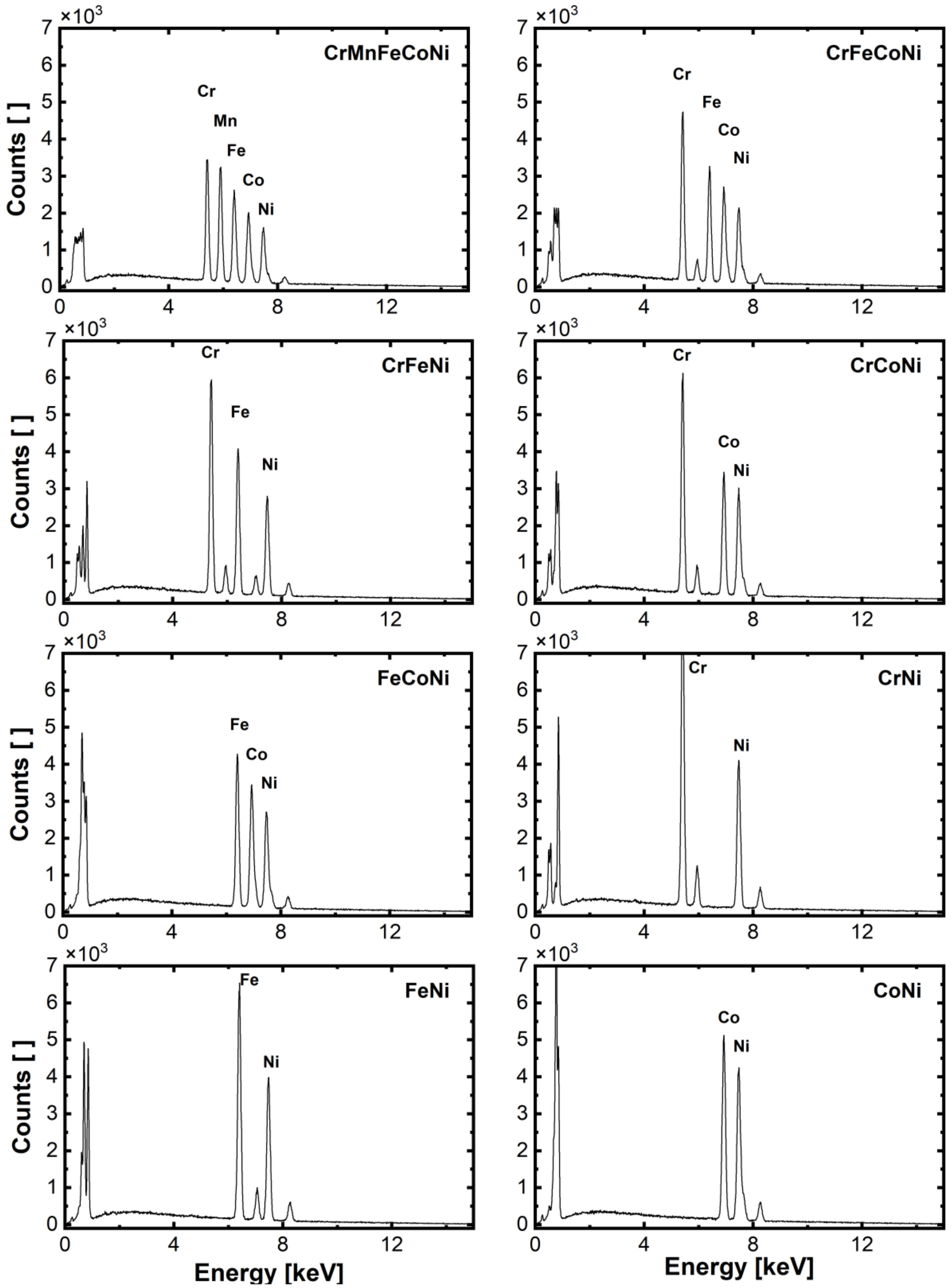

Figure S1: SEM-EDX spectra for Ni-based multi-metallic alloys with assignment of peaks for Cr, Mn, Fe, Co, Ni.

Table S2: XRF analysis of Ni-based alloys.

**Sample**	**Cr (at%)**	**Mn (at%)**	**Fe (at%)**	**Co (at%)**	**Ni (at%)**
CrMnFeCoNi	18.8	18.9	20.9	20.2	21.2
CrFeCoNi	23.6 ± 0.2	--	24.9 ± 0.3	25.9 ± 0.3	25.6 ± 0.3
CrFeNi	32.0 ± 0.3	--	33.8 ± 0.3	--	34.2 ± 0.3
CrCoNi	30.8 ± 0.3	--	--	34.4 ± 0.3	34.8± 0.3
FeCoNi	--	--	32.2 ± 0.2	33.8 ± 0.2	34.0 ± 0.3
CrNi	47.2 ± 0.3	--	--	--	52.8 ± 0.4
FeNi	--	--	49.2 ± 0.3	--	50.8 ± 0.4
CoNi	--	--	--	50.4 ± 0.3	49.6 ± 0.3

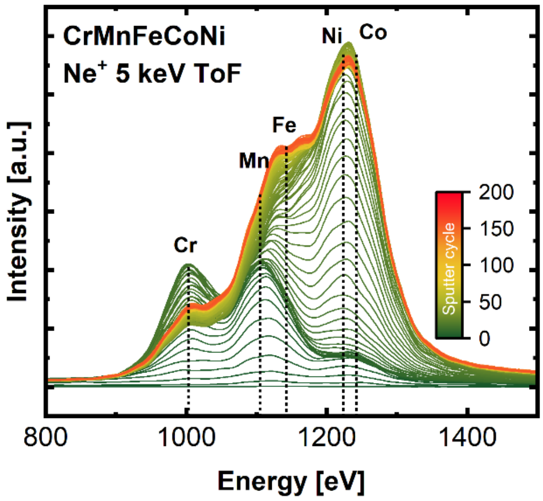

Figure S2: Partial spectra of LEIS sputter profile of CrMnFeCoNi native surface. Peak positions established from reference measurements on monometallic standards are indicated for each element.

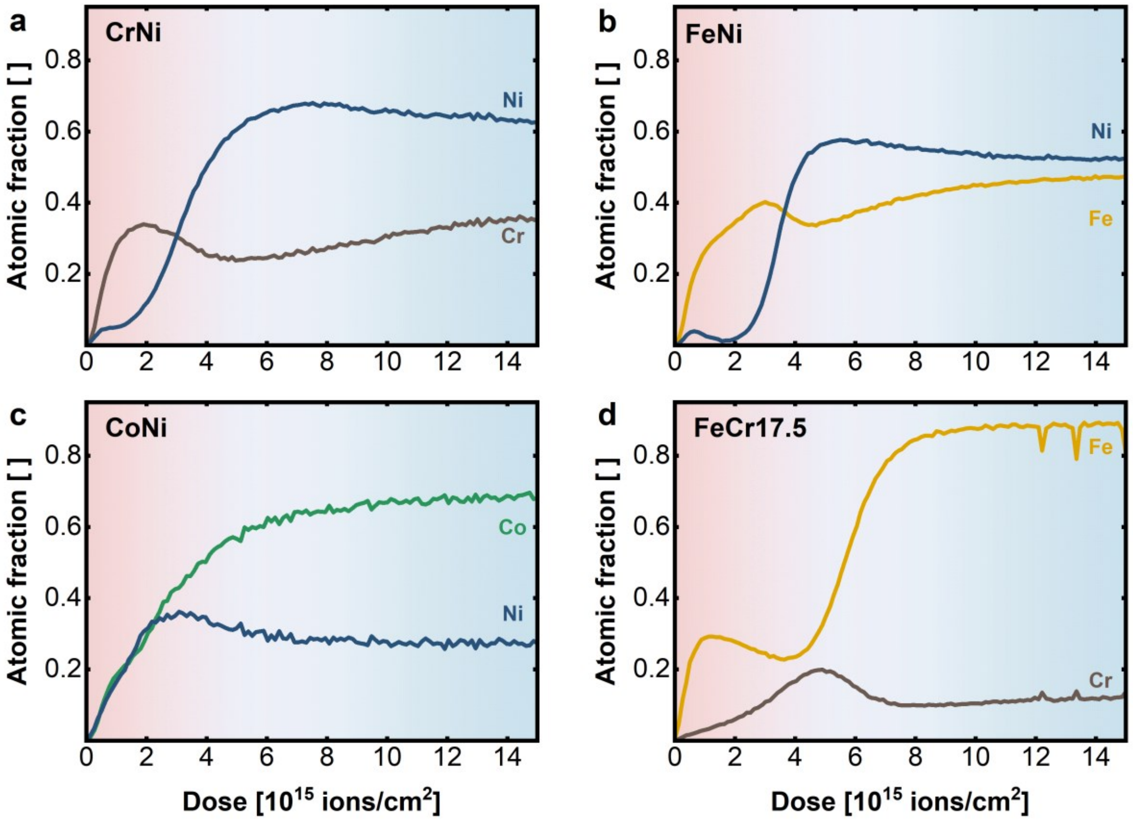

Figure S3: LEIS sputter depth profiles of (a) CrNi, (b) FeNi, (c) CoNi and (d) FeCr17.5.

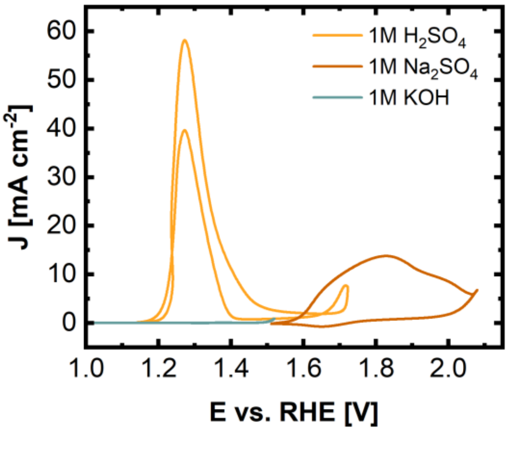

Figure S4: Cyclic voltammograms of CrMnFeCoNi in different conditions at a scan rate of 20 mV s^{-1}

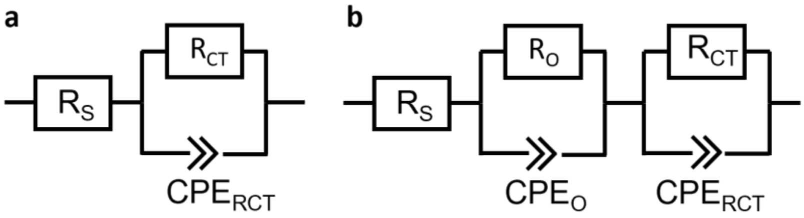

Figure S5: Equivalent circuit used for fitting electrochemical impedance spectra. R_s denotes the electrolyte resistance, R_O the oxide film resistance and R_{CT} the charge transfer resistance. CPE_O and CPE_{RCT} represent the respective constant phase elements.

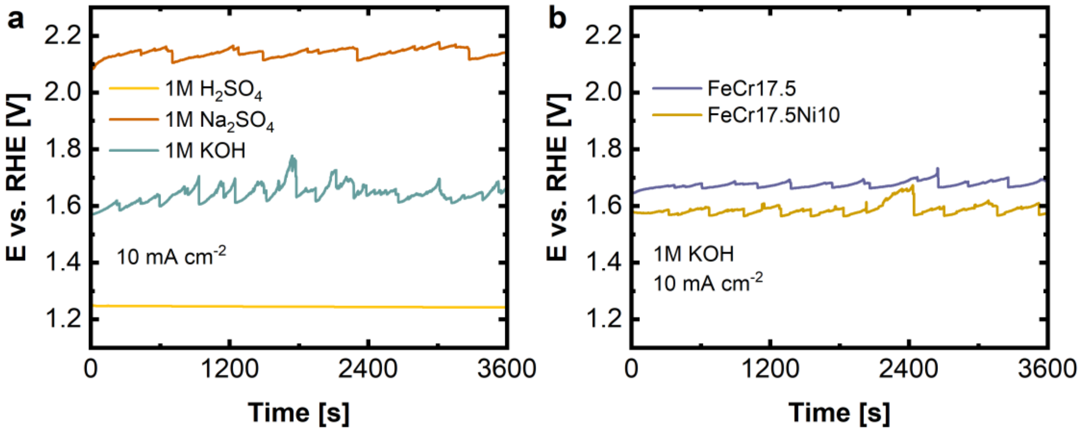

Figure S6: Chronopotentiometric measurements of (a) CrMnFeCoNi in different electrolytes and (b) FeCr17.5, FeCr17.5Ni10 in 1 M KOH. Measurements were performed at a constant current density of 10 mA cm^{-2}.

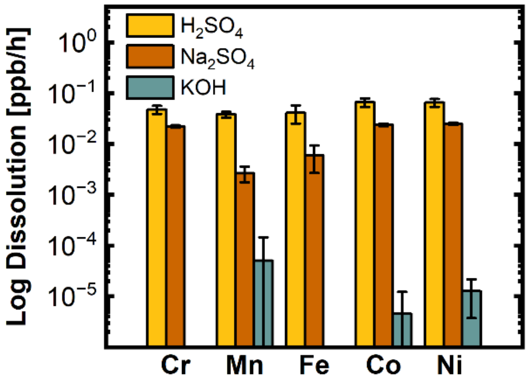

Figure S7*:* Results of the ICP-MS measurements of the electrolyte after chronopotentiometric (CP) measurements of CrMnFeCoNi (in solutions of 1 mM H_2SO_4, 1 mM Na_2SO_4 and 1 mM KOH). Metal dissolution is given in dissolution per hour of CP duration.

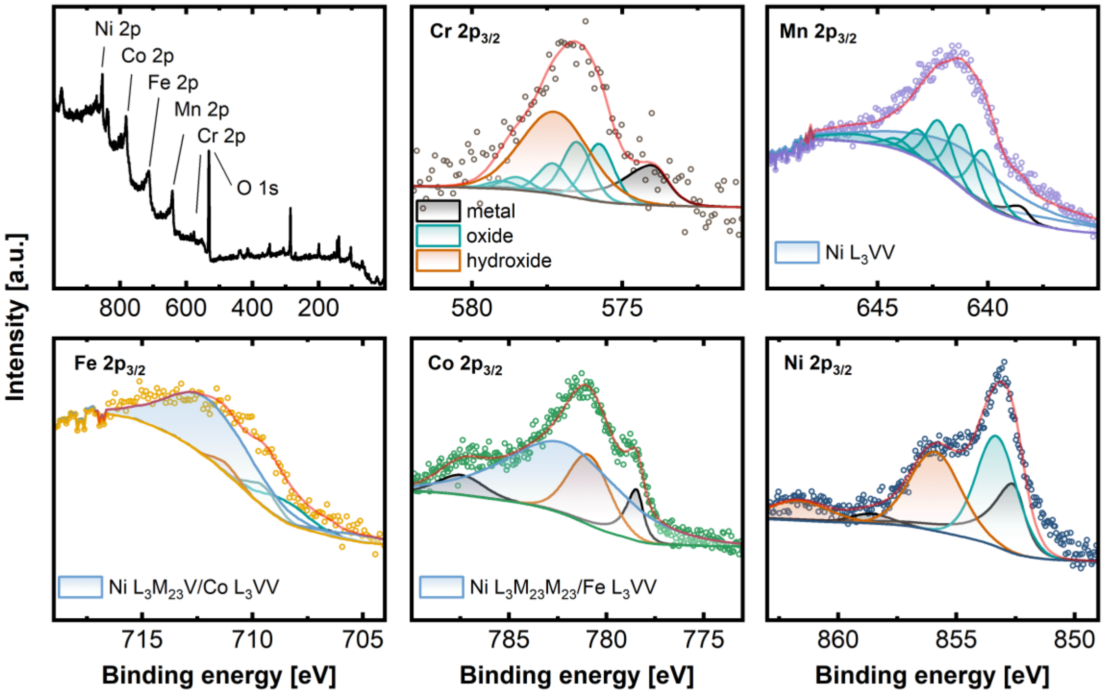

Figure S8: XPS measurements of CrMnFeCoNi after chronopotentiometric treatment for 5.5 h in 1 M KOH at a constant current density of 10 mA cm^{-2}. Recorded a sputter time of 10 s. Survey spectrum as well as high-resolution spectra of the 2p$_{3/2}$ region of Cr, Mn, Fe, Co and Ni.

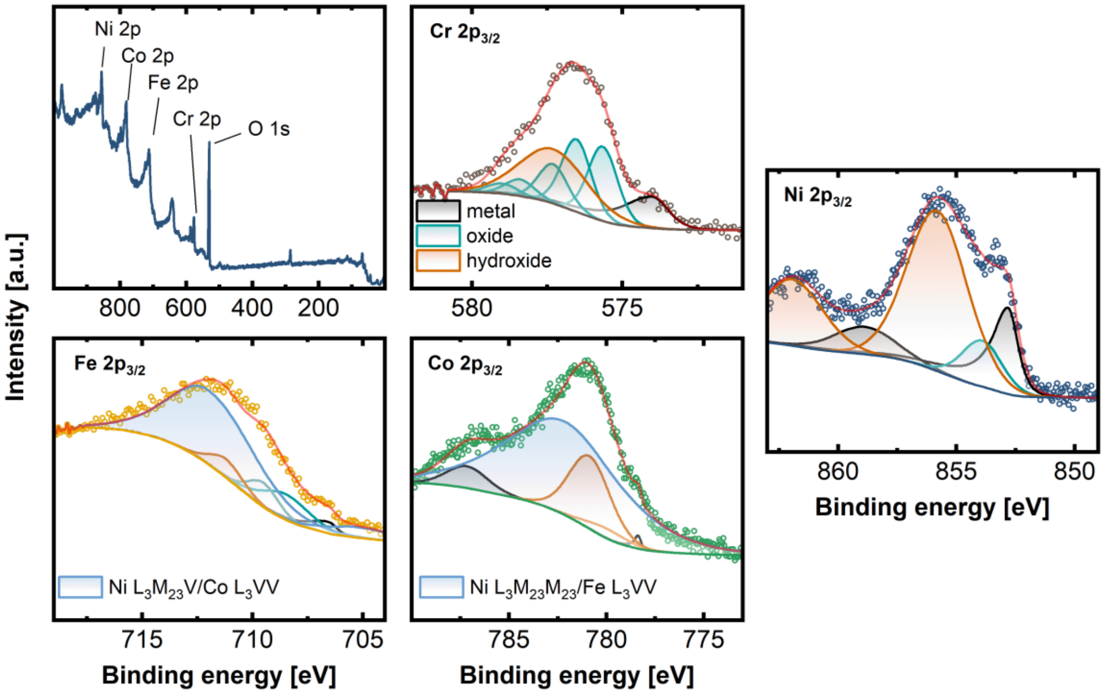

Figure S9*:* XPS measurements of CrFeCoNi after chronopotentiometric treatment for 0.25 h in 1 M KOH at a constant current density of 10 mA cm^{-2}. Recorded a sputter time of 10 s. Survey spectrum as well as high-resolution spectra of the 2p$_{3/2}$ region of Cr, Mn, Fe, Co and Ni.

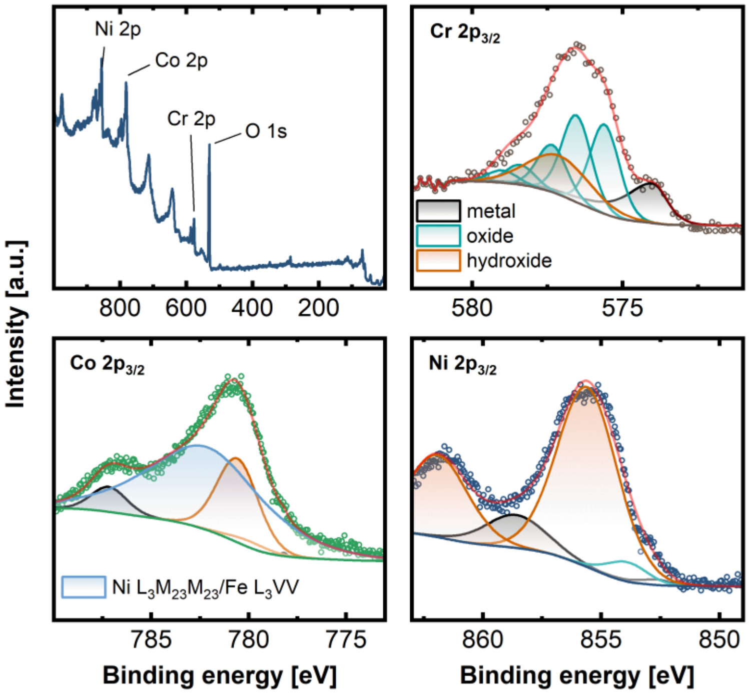

Figure S10: XPS measurements of CrCoNi after chronopotentiometric treatment for 0.25 h in 1 M KOH at a constant current density of 10 mA cm^{-2}. Recorded a sputter time of 10 s. Survey spectrum as well as high-resolution spectra of the $2p_{3/2}$ region of Cr, Mn, Fe, Co and Ni.

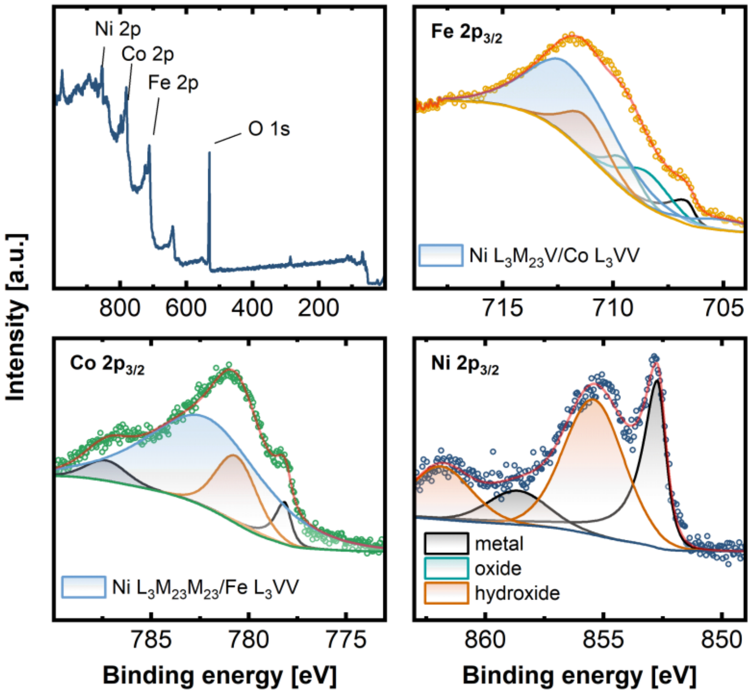

Figure S11: XPS measurements of FeCoNi after chronopotentiometric treatment for 0.25 h in 1 M KOH at a constant current density of 10 mA cm^{-2}. Recorded a sputter time of 10 s. Survey spectrum as well as high-resolution spectra of the $2p_{3/2}$ region of Cr, Mn, Fe, Co and Ni

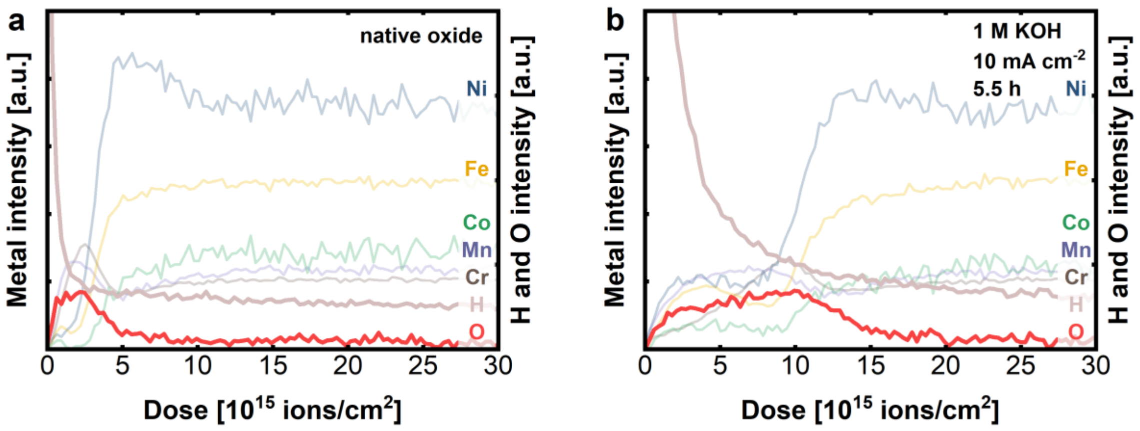

Figure S12*:* LEIS sputter depth profiles of CrMnFeCoNi on (a) the native surface and (b) after chronopotentiometric treatment for 5.5 h in 1 M KOH at a constant current density of 10 mA cm^{-2}. Graphs show qualitative elemental profiles for metals as well as O and H, scaled on different y-axis for better clarity. Metal signals were obtained from deconvolution of Ne 5 keV ToF measurements, whereas O was obtained from He 3 keV ToF spectra. H profile was obtained from He 3 keV Tof measurements, reconstructed from the light sputtered ions signal.

Table S3: EIS fitting parameters using the equivalent circuit shown in Fig. S5a (a) and Fig. S5b (b), respectively.

a

Sample	R_S [Ohm]	Error [%]	R_{CT} [Ohm]	Error [%]
CrMnFeCoNi	33.98	2.549	334.8	1.835
CrFeCoNi	62.48	1.677	415.8	1.794
CrFeNi	59.08	1.335	325.2	1.624
CrCoNi	80.28	1.035	422.6	1.198
FeCoNi	32.71	1.875	781.3	1.599
FeNi	64.54	1.415	346.4	1.116
CoNi	81.2	1.439	207.7	1.858
FeCr17.5Ni10	38.7	1.773	275.7	1.33

b

Sample	R_S [Ohm]	Error [%]	R_O [Ohm]	Error [%]	R_{CT} [Ohm]	Error [%]
CrNi	34.98	0.726	42.85	14.96	125.6	5.482
FeCr17.5	30.75	2.812	76.39	8.46	975.5	0.982

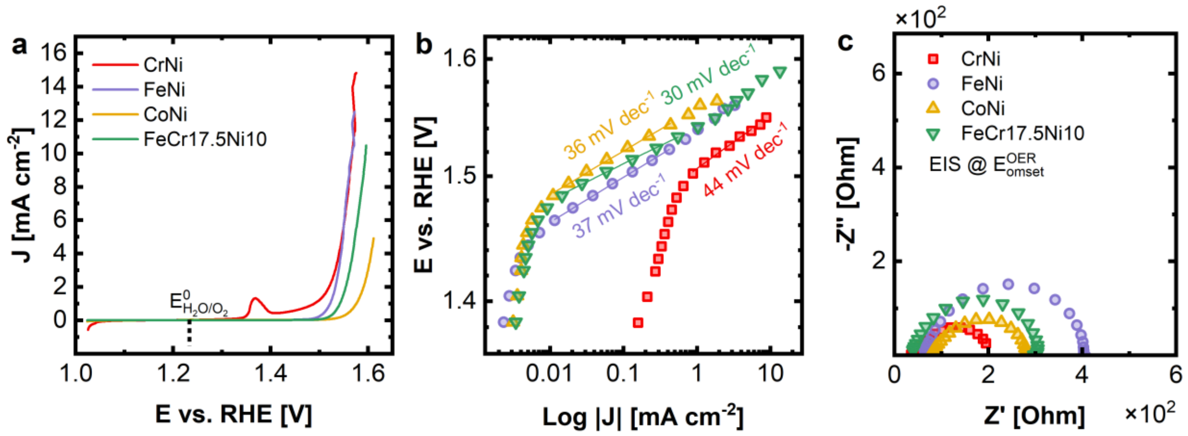

Figure S13: Electrochemical characterization of CrNi, FeNi, CoNi and FeCr17.5Ni10. (a) LSV experiments, (b) Tafel plots with obtained Tafel slopes, (c) EIS spectra performed at the respective OER onset potential.
